# Illuminating the Anticancerous Efficacy of a New Fungal Chassis for Silver Nanoparticle Synthesis

**DOI:** 10.3389/fchem.2019.00065

**Published:** 2019-02-08

**Authors:** Nafe Aziz, Mohd Faraz, Mohd Asif Sherwani, Tasneem Fatma, Ram Prasad

**Affiliations:** ^1^Department of Biosciences, Jamia Millia Islamia, New Delhi, India; ^2^Department of Physics, Indian Institute of Technology Delhi, Haus Khas, New Delhi, India; ^3^Interdisciplinary Biotechnology Unit, Aligarh Muslim University, Aligarh, India; ^4^School of Environmental Science and Engineering, Sun Yat-Sen University, Guangzhou, China; ^5^Amity Institute of Microbial Technology, Amity University, Noida, India

**Keywords:** silver nanoparticles, *Piriformospora indica*, cancer cell lines (MCF-7, HeLa, HepG2), HEK-293, cytotoxic activity, antioxidant activity

## Abstract

Biogenic silver nanoparticles (Ag NPs) have supple platforms designed for biomedical and therapeutic intervention. Utilization of Ag NPs are preferred in the field of biomedicines and material science research because of their antioxidant, antimicrobial, and anticancerous activity along with their eco-friendly, biocompatible, and cost-effective nature. Here we present a novel fungus *Piriformospora indica* as an excellent source for obtaining facile and reliable Ag NPs with a high degree of consistent morphology. We demonstrated their cytotoxic property, coupled with their intrinsic characteristic that make these biogenic nanoparticles suitable for the anticancerous activity. *In vitro* cytotoxicity of biologically synthesized Ag NPs (BSNPs) and chemically synthesized Ag NPs (SNPs) was screened on various cancer cell lines, such as Human breast adenocarcinoma (MCF-7), Human cervical carcinoma (HeLa), Human liver hepatocellular carcinoma (HepG2) cell lines and embryonic kidney cell line (HEK-293) as normal cell lines. The antiproliferative outcome revealed that the BSNPs exhibited significant cytotoxic activity against MCF-7 followed by HeLa and HepG2 cell lines as compared to SNPs. The blend of cytotoxic properties, together with green and cost-effective characteristics make up these biogenic nanoparticles for their potential applications in cancer nanomedicine and fabrication coating of ambulatory and non-ambulatory medical devices.

## Introduction

Cancers are among the foremost reason for mortality as per WHO (World Health Organisation) report 2018. About 1.7 million new cases are expected to be diagonised in 2018 and around 609,640 cancer deaths were estimated in United State by 2018 (American Cancer Society, 2018). Cancer death rates are the best measures to detect progress rate against cancer as they are less affected by detection practices than incidence and survival. Cancer usually develops with older age group; In the United State 87% of all cancers are detected in the age group of 50 years or more (American Cancer Society, 2018). Cancer causing behaviors such as smoking, eating an unhealthy diet, and not being physically active also increases risk of getting cancer (Ahmedin et al., 2011; Ferlay et al., 2012; Siegel et al., 2016; Zeng et al., 2018). In United State alone 40 out of 100 men and 38 out of 100 women develop cancer during their lifetime (American Cancer Society, 2018). The primary cause of cancer is an abnormal growth of cells caused by variations in the gene expression leading to an uncontrolled cell proliferation, which invades and infects the distant tissues (Balakumaran et al., 2015). Many techniques such as chemotherapy, immunotherapy, and radiotherapy have been used for the treatment of cancer (Kedar et al., 2010; Chen and Kuo, 2017). Conventional chemotherapy interfere with DNA synthesis along with mitosis, causing death of active cancer cells, but these techniques are unable to minimize the harmful effects on the healthy tissues causing adverse side effects, e.g., loss of appetite and nausea, leading to death of the cancer patients (Lee et al., 2007; Senapati et al., 2018). Additionally, the bio-accessibility of these drugs to tumor tissues is fairly poor, and require higher doses, leading to high toxicity in normal cells and also leeds to multiple drug resistance (Kato et al., 1995; Lupu et al., 1996; Johnston, 1997; Brown, 2002). Therefore, it is desirable to develop biocompatible and profitable methods that can either passively or actively target cancerous cells, thereby reducing adverse side effects while improving therapeutic efficacy. Biosynthesized nanoparticles have paved the way and attracted researchers across the world due to their specific properties and wide applications in biomedical sciences (Shin et al., 2009; Zhou et al., 2011; Aziz et al., 2015, 2016; Rajpal et al., 2016a; Khan et al., 2017). Silver nanoparticles (Ag NPs) have potential to treat a variety of diseases, such as retinal neovascularisation (Ong et al., 2013) and have been investigated among the emerging nanoproducts and novel cancer therapeutics.

To meet the extensive range of nanoparticles, a number of methods have been exploited such as physical, chemical, and biological methods (Prasad et al., 2016). Out of these, physical methods suffer a setback of low yield of nanoparticles (Malik et al., 2002), while the chemical methods require toxic chemicals for reduction of metallic ions to nanoparticles, which further lead to generation of hazardous by-products (Prasad, 2014). On the other hand biological synthesis of Ag NPs through bottom up approach using various resources (e.g., plants and its products, fungi, algae, and bacteria, etc.) are cost effective, eco-friendly, least toxicity, less tedious, high yield, and most importantly their biocompatibility with high reduction potential (Thakkar et al., 2010; Lemire et al., 2013; Aziz et al., 2014; Prasad et al., 2016). Size and shape of these Ag NPs can be easily modified using some parameters such as pH and temperature (Bhattacharya and Mukherjee, 2008; Ngo et al., 2011; Aziz et al., 2015) and do not require any additional stabilizer in order to prevent aggregation, as the cellular proteins themselves serve as stabilizer (Aziz et al., 2015). The global intensification of antibiotic resistance and the lack of discovery of new antibiotics in the market have led to the research on nanostructured material-based antimicrobial therapy that has now moved toward clinical studies (Arvizo et al., 2012; Aziz et al., 2016).

According to the previous studies, Ag NPs causes cytotoxicity of the cancer cells (Yoon et al., 2007) by altering the morphology, reducing the viability along with oxidative stress in glioblastoma and fibroblast cells (Asharani et al., 2009), human and rat liver cells (Hussain et al., 2005; Kim et al., 2010), HeLa cells (Sonoda et al., 1998), and THP-1 monocytes (Hsin et al., 2008; Foldbjerg et al., 2009). Ag NPs shown positive results as anticancer agents (Singh and Ramarao, 2012) which opens new entries toward the field of nanomedicines. Ag NPs were also found to play an effective role in tumor control via their cytotoxic effects (Sukirtha et al., 2012). Currently, an array of cytotoxic agents have been employed to treat cancers, but their efficacy and demerits are uncertain (Franco-Franco-Molina et al., 2010). Thus, there is a need to develop a novel, profitable and biocompatible therapeutic agents against cancer.

In view of problems discussed above, the current study deals with the biosynthesis of smartly tailored Ag NPs using biogenic approaches and their potential to evaluate toxicity against cancer. We have established the cytotoxic efficiency of BSNPs derived from fungal chassis *P. indica* order Sebacinales. This is the first report employing the endophytic root colonizing fungus for Ag NPs synthesis and it exhibit high efficiency in growth with less cultivation time. Importantly, we study that these Ag NPs and compared with chemically synthesized silver nanoparticles (SNPs), demonstrated competitive anticancerous activity when incubated with different concentrations of silver nanoparticles. Our result offers an alternate platform for the utilization of Ag NPs as anticancerous agent which lay concrete on the way for the animal model studies.

## Materials and Methods

### Reagents

All the reagents taken were of analytical grade from Sigma-Aldrich, USA, Merck and Hi-Media, India. Double distilled water was used throughout the experiments.

### Microbial Culture and Extract Preparation

*Piriformospora indica* was procured from Amity University, Noida, India and culture in MYP medium containing 7.0 g/L malt extract, 0.5 g/L yeast extract and 1.0 g/L peptone at pH 6.1 ± 0.2 and incubated at 180 rpm and 28 ± 2°C for 7 days.

The fungal biomass was separated out through filtration using cheese cloth, washed thrice with double distilled water (ddH_2_O). Ten gram of wet fungal biomass and were suspended in 100 mL of ddH_2_O for 72 h at 180 rpm and 28 ± 2°C. The mycelia were separated by filtration using Whatman filter paper No. 1 and filtrate was used as aqueous extract.

### Nanoparticles Characterization

Thirty milli liter of the extract mixed with 70 mL of ddH_2_O, along with the addition of 1 mM silver nitrate (AgNO_3_) in 250 mL conical flask at 180 rpm and 28 ± 2°C in dark. A control without AgNO_3_ was also taken having similar condition. As the solution turned reddish brown due to the reduction of AgNO_3_ to Ag NPs the color intensity was measured using UV-Vis Spectrophotometer. The bio reduction of AgNO_3_ to Ag NPs was examined using double beam UV-vis spectrophotometer (UV-1800, Shimadzu, Japan) periodically to access change in the optical property up to 96 h. After completion of the reaction the sample were centrifuge at 15,000 rpm for 20 min. Before further characterization the Ag NPs were purified by separating the unwanted biological molecules (Premasudha et al., 2015). Pellet was redispersed into 1 mL of deionized water and sonicate for 5 min to ensure proper dispersion for washing (Kayalvizhi et al., 2014). Centrifugation, re-dispersion and sonication were repeatedly carried out followed by lyophilisation to obtain powdered nanoparticles.

Scanning electron microscopy (SEM) (Zeiss Evo HD, Jena, Germany) was done along with the energy dispersive X-ray (EDAX) (EDX, 10 mm^2^ SDD Detector-X-act, Oxford Instruments, Oxfordshire, United Kingdom) with an acquisition time ranging from 60 to 100 s and the accelerating voltage of 20 KV and TEM (Philips, EM-410LS, JEOL, Japan) for the surface morphology and the presence of elemental silver present inside the biologically synthesized nanoparticles (BSNPs). Sample for electron microscopy was prepared by taking 1 mg of BSNPs in 1 mL of ethanol and sonicate it for 15 min. Sample (10 μL) was spread on the grid and dried it at room temperature. Further sample was analyzed after coating it with gold. FTIR analysis (Varian 7000 FTIR, California) was done by making KBr pellet with BSNPs for vibrational structural characterization. Measurements were performed in 650–4,000 cm^−1^ range at a resolution of 4 cm^−1^. XRD (Rigaku, Ultima IV, Japan) of the powdered BSNPs, SNPs as well as cell extract was performed and the data were recorded in the 2θ range of 20°–80° having K-beta filter with X-Ray 1.54056 Å at 40 kV and 30 mA.

### Cell Recovery

HEK-293, MCF7, HeLa, and HepG2 cell lines were obtained from NCCS, Pune, India. Cultured in T-25 tissue culture flasks with DMEM (Dulbecco's Modified Eagle's Media) with 10% fetal bovine serum, and antibiotics (100 U/mL), and incubated at 37°C with 5% CO_2_.

### Antioxidant Activity

Antioxidant activity of BSNPs was determined through 1,1-diphenylpicrylhydrazyl (DPPH) method. BSNPs at a stock concentration of 50 μg/mL was taken, different dilution (5, 10, 15, and 20 μg/mL) was made in methanol. Methanolic DPPH solution (1 mL) at a concentration of 0.3 mM was added to 3.0 mL samples of various concentrations. Incubate the reaction mixture at room temperature for 30 min absorbance was taken at 517 nm. The scavenging potential of samples was calculated by using following formula:

(1) $$\begin{array}{r} \%\text{ of inhibition}=\frac{\text{A control }-\text{ A Sample}}{\text{A control}}\times 100 \end{array}$$

A_control_ (L-ascorbic acid) as standard and A_sample_ as absorbance of samples taken. The methanolic DPPH solution (1 mL, 0.3 mM) was used as a control.

### Cell Viability Assay

Cell viability assay was performed using the technique described by Al-Fatlawi and Ahmad (2014). It is a colorimetric assay to measure growth of the cells which reduces the tetrazolium yellow dye, called MTT, to insoluble purple color formazan using mitochondrial dehydrogenase enzyme of living cells. The absorbance was measured at 570 nm wavelength. Cytotoxic potential of the Ag NPs was determined by dose dependent manner. RPMI-1640 culture medium with 10% heat-inactivated fetal calf serum along with antibiotic antimycotic solution was used to culture cancer (MCF7, HeLa and HepG2) as well as normal cell lines (HEK-293). Cells were plated in 96-well plate having a density of 5 × 10^3^ cells per well and cultured for 24 h at 37°C. BSNPs and SNPs stock solution were prepared in a 1:1 mixture of DMSO (Dimethyl Sulfoxide) and THF (Tetrahydrofuran) and cells were incubated for 48 h and cell proliferation was calculated by adding 20 μL of MTT dye (5 μg/mL in phosphate-buffered saline) per well. Further the plates were placed for 4 h at 37°C in a humidified chamber containing 5% CO_2_. Due to reduction of the tetrazolium dye, crystals of formazan formed by viable cells in each well were dissolved in 150 μL dimethyl sulfoxide and absorbance was measured at 570 nm. The absorption values were expressed as cell viability (%), according to the control group as 100%. Doxorubicin (Doxo) and 5-Fluorouracil (5-Fu) were used as standard drugs. The assays were carried out in triplicate on three independent experiments. The concentration required for 50% inhibition of cell viability (IC_50_) was calculated using the software “Prism 3.0.”

### Statistical Analysis

Graph Pad Prism was used for data analysis. The mean and standard deviation were used for descriptive statistical measures to summarize the data. To evaluate the influence of independent variables as well as possible interactions between them in the cytotoxicity efficacy study, two-way analysis of variance (two-way ANOVA) was used. Tukey's procedure was determined, whether the data showed evidence of the difference between the various cytotoxic agents.

## Results

### Fungal Derived Ag NPs Synthesis and Characterization

**Figure 1:**
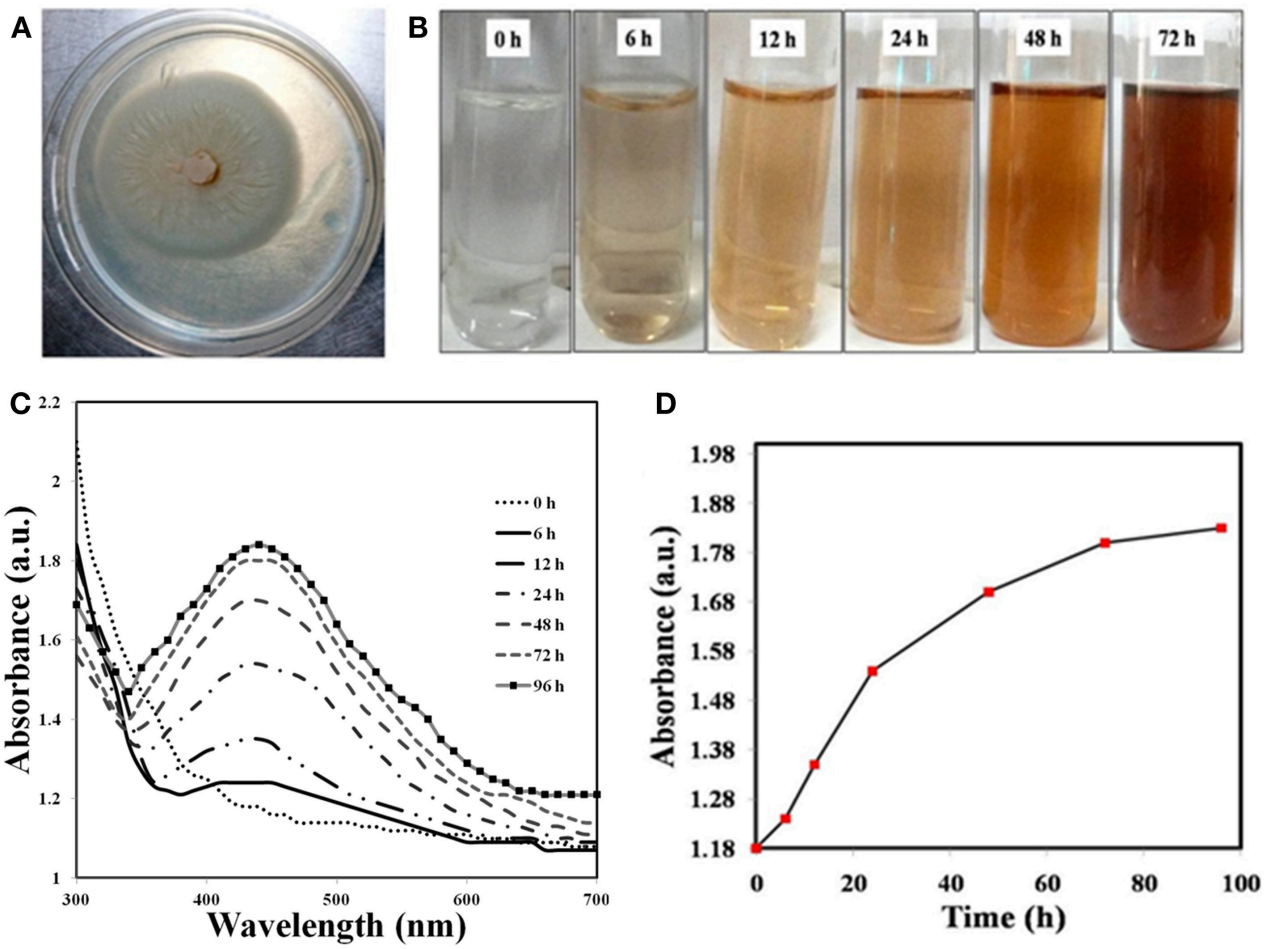
Optical Properties. **(A)** Colony morphology, **(B)** Optical images of biosynthesized Ag NP show a range of vibrant colors from colorless (start of biosynthesis) to light brown and, finally, yellowish brown after 72 h. **(C)** UV-vis spectra of *P. indica* derived Ag NPs at various reaction times; **(D)** Reaction saturation curve indicating the evolution of the Plasmon band as a function of time.

**Figure 2:**
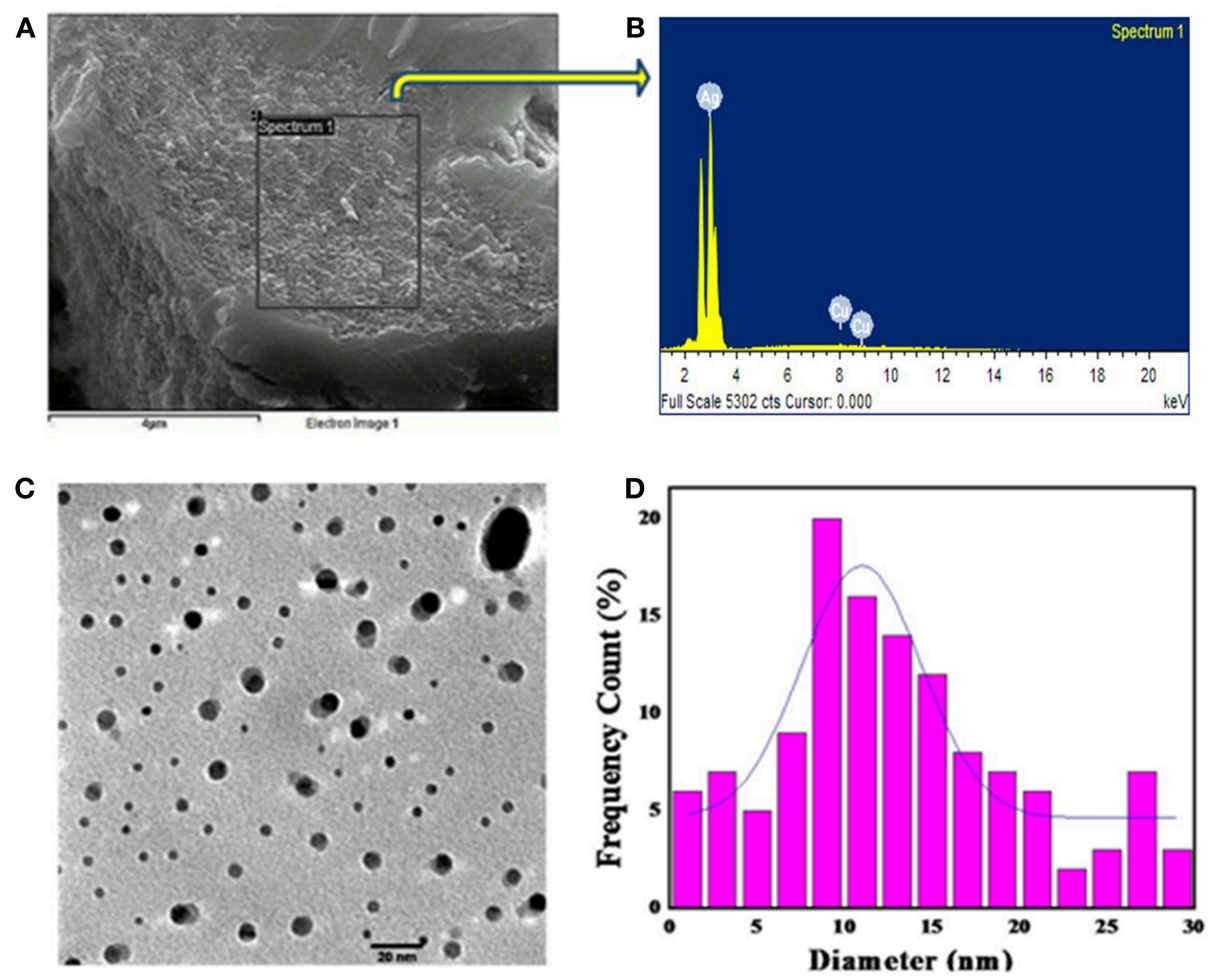
Morphological characterization of *P. indica-* derived Ag NP. **(A)** SEM image of biogenic Ag NPs (scale bar indicates 4 μm); **(B)** EDX spectrum of biogenic Ag NPs; **(C)** TEM image of the biogenic Ag NPs (scale bar indicates 20 nm); **(D)** Histogram of the size distribution of biogenic Ag NPs.

**Figure 3:**
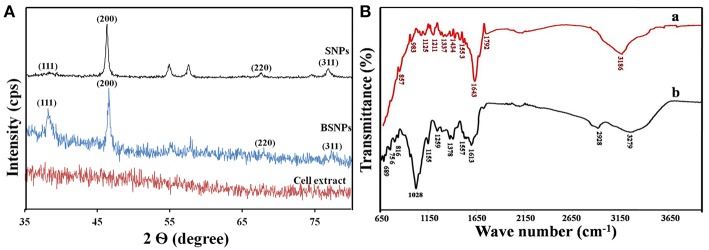
Structural and biochemical characterization. **(A)** XRD spectrum showing the face centered cubic (FCC) nature of the SNPs, BSNPs and cell extract. **(B)** FTIR spectra of (a) BSNPs and (b) cell extract.

The growth characteristics of the fungus show that mycelia grown in MYP agar media were white, flat and submerged (Figure 1A). To test our hypothesis, we incubated the cell extracts with 1 mM AgNO_3_ solution and observed a steady color change from colorless to reddish-brown up to 96 h (Figure 1B). UV-vis absorption of the BSNPs feature at 440 nm (Figure 1C) and the color intensity persisted even after 72 h, which signifies that particles were well-dispersed and stable in the solution. As seen in Figure 1D, the intensity of the peak increases with time before reaching a saturation point. Figures 2A,B illustrates SEM image along with EDAX of the BSNPs. The elemental silver was found more than 80% of the weight (Table 1) and the percentage of carbon, oxygen and copper are in less amount. The particles size distribution was determined with TEM (Figures 2C,D), and observed to be fairly consistent in the range of 6–15 nm with only small fraction of the particles having dimensions 16–30 nm. The XRD pattern of the BSNPs and SNPs along with the cell extract are illustrated in Figure 3A. Four peaks of BSNPs at 2θ values of 38.0, 46.4, 67.8 and 77.2 degrees corresponding to (111), (200), (220), and (311) planes of silver were observed which are consistent when compared with the standard powder diffraction card of the Joint Committee on Powder Diffraction Standards, silver file No. 04–0783 (Yang et al., 2011). The XRD observations consequently verified that the obtained BSNPs and SNPs were face centered cubic (FCC), thus confirming that BSNPs and SNPs were crystalline in nature. The average size (D) was estimated using the Debye-Scherrer formula:

(2) D=0.9λβ cosθ

Where “λ” represent X-Ray wavelength (0.1541 nm), “β” is the line broadening at, half the maximum intensity (FWHM) and “θ” is the Bragg's angle. Based on the different 2θ values, we calculated a range of average crystalline size (D) between 5 and 20 nm with an average of ca. 13 nm consistent with the previous TEM as stated in Figure 2D. Where as for SNPs the average crystalline size between 10 and 25 nm.

**Table 1:** EDAX analysis of biogenic silver nanoparticles.

**Element**	**Weight %**	**Atomic %**
C	7.45	31.11
O	9.46	29.66
Cu	1.87	1.48
Ag	81.22	39.23

Figure 3B shows the FTIR spectra of the possible biomolecules present in the cell extract and the BSNPs. The spectrum of the extract with peak pick at 3,279, 1,613, and 1,028 cm^−1^ which are attributed to the OH stretching vibration of the aromatic compounds (e.g., Phenols), NH_2_ stretching of the primary amines, respectively and lastly 1,028 cm^−1^ correspond to C-O of the ether group and C-N stretching of the amine group. The bands at 2,928, 1,378, and 816 cm^−1^ indicated CH stretching and bending of the alkanes and C–H bending of the alkenes, respectivel**y** providing evidence of the presence of hydrocarbons. Furthermore, the bands at 1,557, 1,259, and 1,155 cm^−1^ represented C = C stretching of the aromatic compounds, P = O stretching of the phosphorous containing groups, and the last one represented the phosphate, respectively. Whereas, the FTIR spectrum of the BSNPs was less intense and broadened. The peak at 2,928 cm^−1^ corresponding to C–H stretching of the alkanes associated with hydrocarbons was absent. The C = O and CH vibration of amide and amine groups are shifted. Reduction in the intensity of 1,028 cm^−1^ peak after biosynthesis of BSNPs suggested that the protiens were involved in synthesizing and capping of nanoparticles. The additional peaks at 1,643, 1,211, 1,337, and 1,792 cm^−1^ corresponded to stretching band of alkenes and N-H bending, C-O stretching vibration for ether group, C-N stretching of aromatic amines and lastly for stretching vibration of C = O of conjugated acid halide, respectively.

### *In vitro* Antioxidant and Cytotoxic Activity

**Figure 4:**
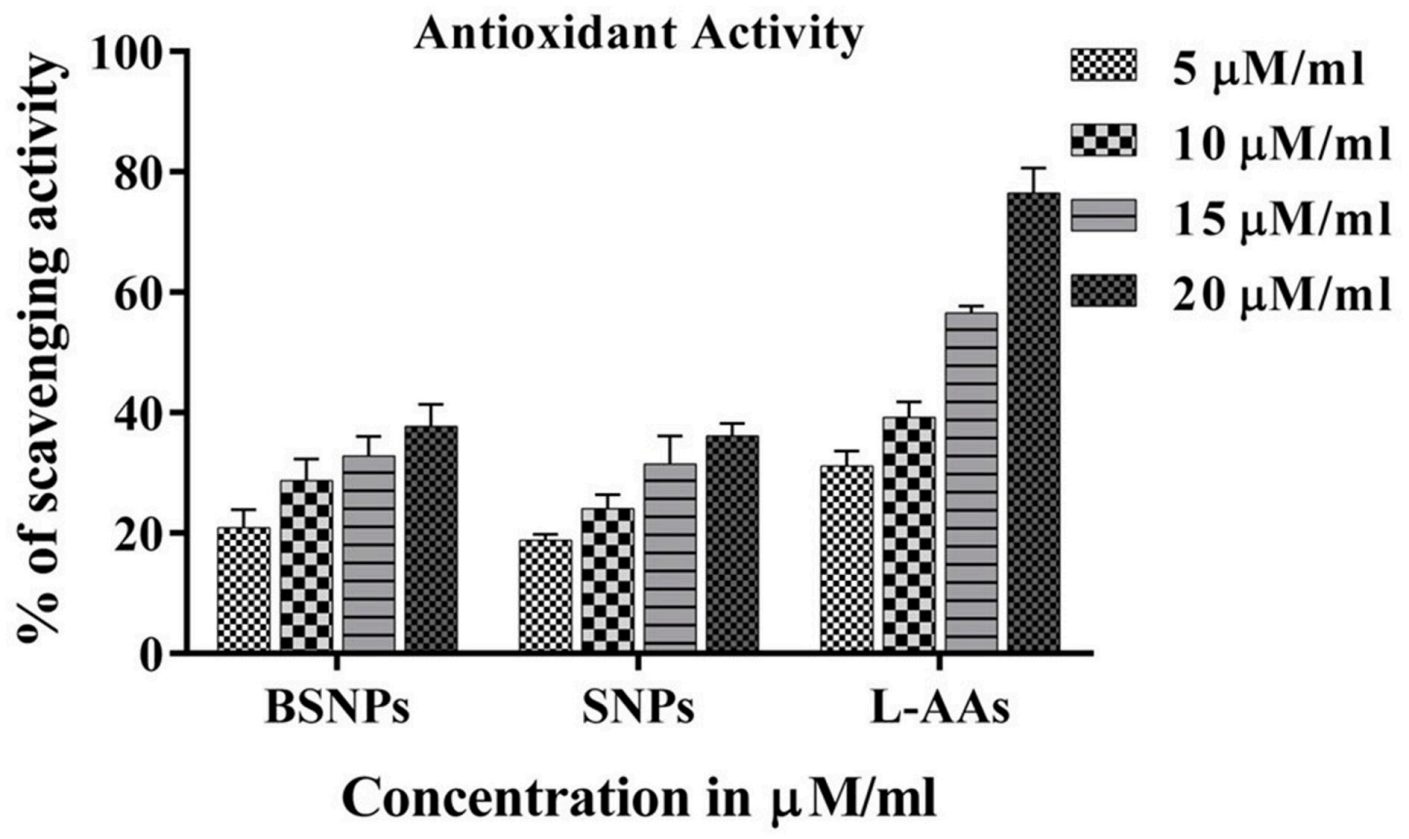
The scavenging potential data of biogenic silver nanoparticles (BSNPs) and chemically synthesized silver nanoparticles (SNPs). The values demonstrate the mean ± standard error mean (SEM) % scavenging activity of the compounds using L-ascorbic acid (Standard). Experiments were performed in triplicates, the error bar represents statistically significant (*p* < 0.05).

**Figure 5:**
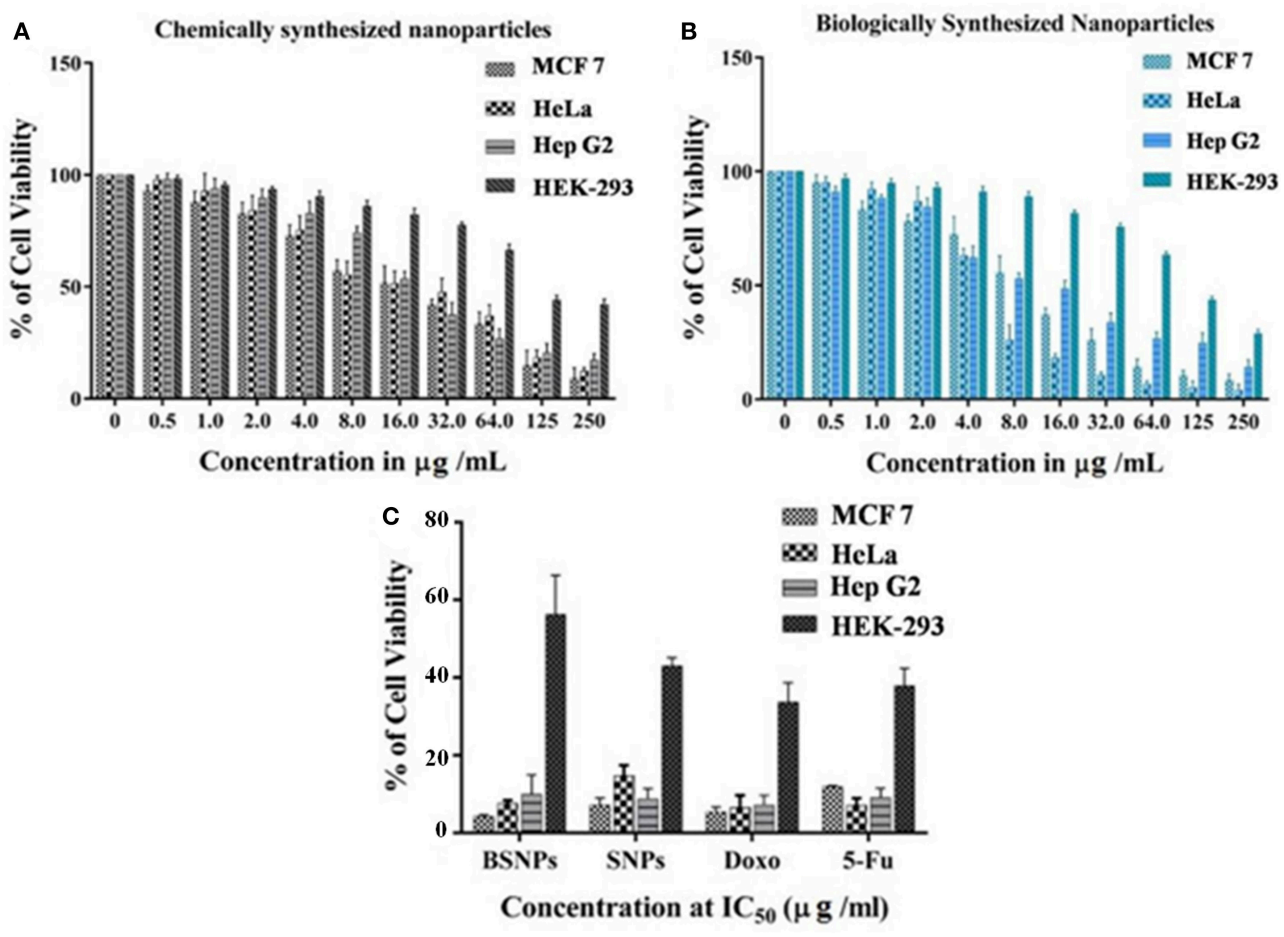
Experimental observation of the cytotoxic property of *P.indica* -derived Ag NPs, **(A,B)** cytotoxic activity of chemically Ag NPs and biogenic synthesized Ag NPs at different concentration against cancer cell lines. Experiments were performed in triplicates; the error bar represents statistically significant differences (*p* < 0.05). **(C)** Cytotoxic activity (IC_50_) of biogenic silver nanoparticles (BAgNPs) and chemically silver nanoparticles (SNPs) against human carcinoma cell lines and normal cells (HEK-293). Doxorubicin (Dox) and 5-Fluorouracil (5-Fu) are used as reference drugs.

**Figure 6:**
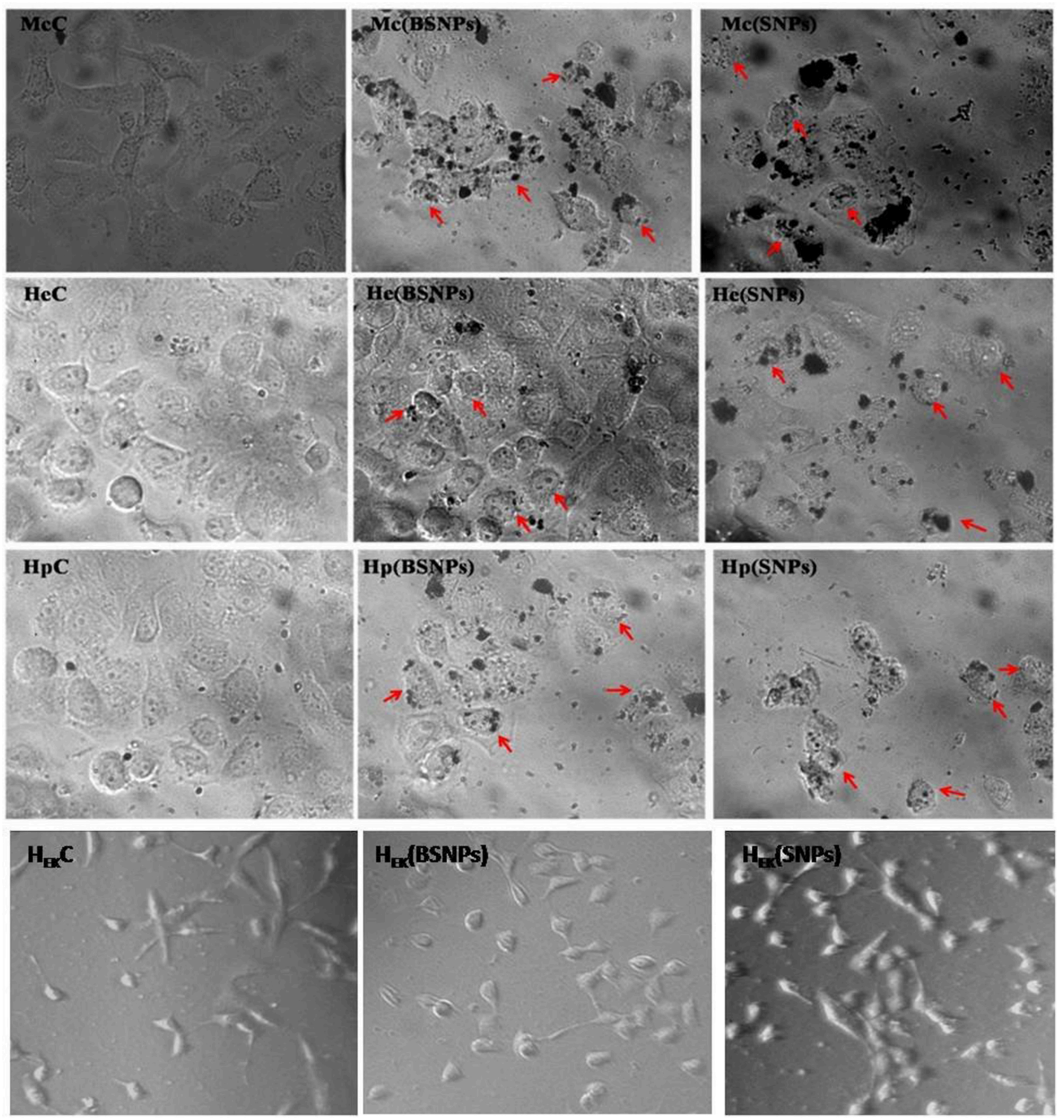
Cytotoxic activity of biogenic synthesized silver nanoparticles at IC_50_ against different cancer cell line Mc (MCF 7), He (HeLa), Hp (HepG2), H_EK_ (HEK-293), C (control), BSNPs (Biogenic Silver nanoparticles) and SNPs (Chemically synthesized silver nanoparticles).

The antioxidant activity of the BSNPs at different concentrations (5, 10, 15, and 20 μg/mL) was shown in Figure 4 and Table 2. The results revealed that the BSNPs are almost similar antioxidants in comparison to the chemically synthesized Ag NPs (SNPs). The effect of BSNPs in different dose on cell viability of diverse cancer cell lines MCF7, HeLa, and HepG2 along with HEK-293 as normal cell lines showed an increment in cytotoxicity with an increase in the concentration of Ag NPs (Figure 5). The cytotoxicity potential of the BSNPs and SNPs against cancer cell lines was IC_50_ < 3.60 μg/mL (Figure 6 and Table 3) whereas for normal cell cytotoxicity potential was IC_50_ > 50 μg/mL.

**Table 2:** Scavenging potential data of biogenic silver nanoparticles (BSNPs) and chemically silver nanoparticles (SNPs).

**Compounds**	**5 μg/mL**	**10 μg/mL**	**15 μg/mL**	**20 μg/mL**
Biogenic silver nanoparticles (BSNPs)	19.6 ± 1.2	27.2 ± 1.3	31.4 ± 1.5	36.1 ± 1.4
Chemically silver nanoparticles (SNPs)	18.3 ± 1.48	23.1 ± 1.3	29.4 ± 1.4	35.3 ± 1.4
L-ascorbic acid	32.3 ± 1.3	38.6 ± 0.91	57.5 ± 1.4	77.3 ± 1.3

*Result were expressed as mean ± standard error mean % scavenging activity of the compounds using L-ascorbic acid (Standard)*.

**Table 3:** Cytotoxic activity (IC_50_ ± SD values) μg/mL of biogenic silver nanoparticles and chemically synthesized silver nanoparticles against human carcinoma cell lines and normal cells (HEK-293).

**Compounds**	**MCF7**	**HeLa**	**HepG2**	**HEK-293**
Biogenic silver nanoparticles (BSNPs)	1.07 ± 0.19	1.87 ± 1.31	2.45 ± 0.62	13.94 ± 1.31
Chemically silver nanoparticles (SNPs)	1.67 ± 0.10	3.55 ± 0.94	2.09 ± 1.21	10.33 ± 0.77
Doxo	1.28 ± 0.20	1.46 ± 0.24	1.87 ± 0.20	7.98 ± 1.21
5-Fu	2.95 ± 0.10	1.70 ± 0.72	2.26 ± 0.91	9.12 ± 1.32

*Reference drugs are used as Doxorubic (Dox) and 5-Fluorouracil (5-Fu)*.

The experimental IC_50_ data revealed that the concentration of BSNPs were relatively more cytotoxic than SNPs with significant IC_50_ values of 1.07 ± 0.19 μg/mL (MCF7), 1.87 ± 1.31 μg/mL (HeLa) and 2.45 ± 0.62 μg/mL (HepG2) in all cancer cell lines. Whereas, SNPs exhibited maximum inhibitory effect against MCF7 (1.67 ± 0.10) μg/mL then HepG2 (2.09 ± 1.21) μg/mL lastly in HeLa (3.55 ± 0.94) μg/mL at IC_50_ values. Comparatively, the IC_50_ values were much higher in normal cell (HEK-293) which indicate that normal cells were almost unaffected by BSNPs. So, among the all tested cancer cell lines the most effective cytotoxicity was found against MCF7 followed by HeLa and HepG2 cell lines against BSNPs.

## Discussion

**Figure 7:**
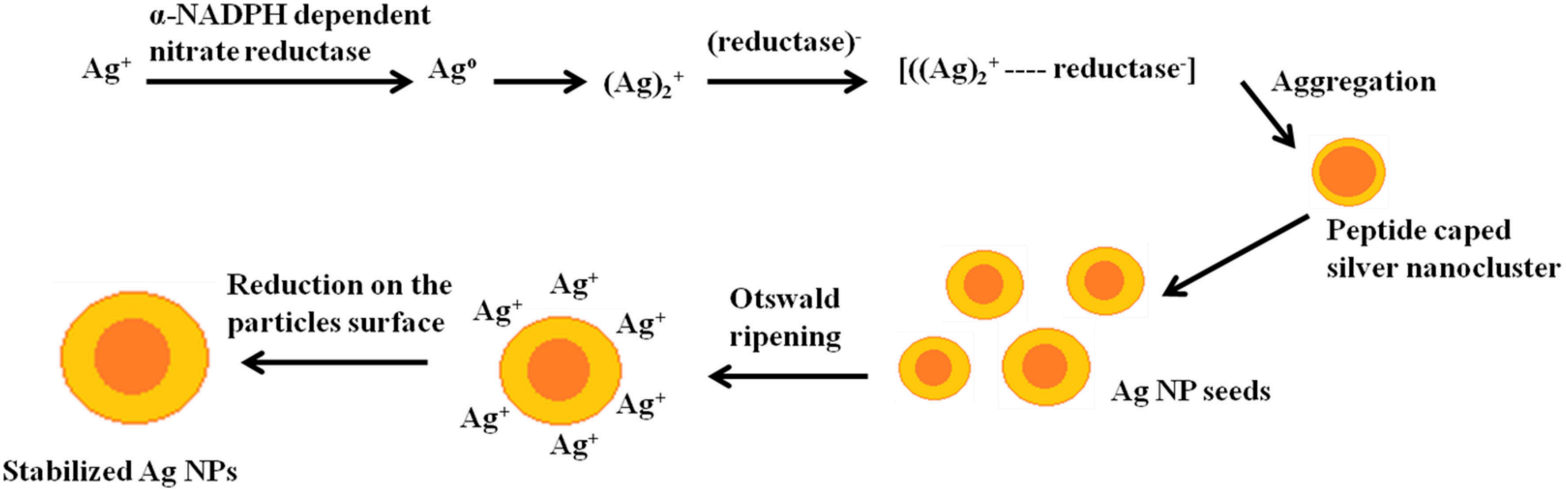
Representation of the reduction and formation of stabilized Ag NPs.

*P. indica*, is a root entophytic fungus and a member of order Sebacinales. It colonizes in most of the members (bryophytes, pteridophytes, gymnosperms, and angiosperms) through mycorrizal interactions. Root colonization results into significant increase in the plant growth, early flowering, higher seed yield, alteration in the secondary metabolites (Varma et al., 1999). *P indica* also shows enormous bio-protective potential against plant pathogens and insect pests of agricultural and horticultural crops (Varma et al., 2012). It is well-known for producing various types of enzymes like urease, enolase, glutamate dehydrogenase and valuable source for phosphate solubilizing plant growth promoting factors and α- NADPH dependent nitrate reductase (Prasad et al., 2013; Gill et al., 2016; Siddhanta et al., 2017). As expected, the silver nanoparticles were produced when the extract seized the Ag^+^ ions present inside the solution containing AgNO_3_ into their elemental form (Ag°) by utilizing cellular extract for example NADPH-dependent nitrate reductase as illustrated in the Figure 7 (Prasad et al., 2016). The enzymes and the other reducing agents present inside the cell extract are beneficial for large-scale nanoparticle synthesis and its isolation (Ray et al., 2011). The visual observations were substantiated by longitudinal excitation of surface Plasmon in BSNPs (Pandey et al., 2012). Earlier studies showed that NADPH-dependent nitrate reductase and shuttle quinine of *Fusarium oxysporum* are responsible for synthesis of nanoparticles (Prabhu and Poulose, 2012). The morphological characterizations through SEM with EDX showed that, the percentage of BSNPs synthesized were high, which signified the purity of the compound. The percentage of Copper and Carbon might be due to carbon coated copper grid used during sample preparation. Carbon and oxygen are sign of microbial biomolecules present on the surface of Ag NPs through EDAX. Carbon and oxygen in the samples confirm the presence of stabilizers composed of alkyl chains (Kaushik and Joshi, 2015; Aziz et al., 2016). Further TEM and XRD studies shown that BSNPs were spherical shape with polydispersed in nature and confirmed the crystallinity of the particles, similar results were shown by Aziz et al. (2015).

The enzymes and proteins secreted by the microorganisms impart better stability to Ag NPs which reduce the requirement of downstream processing of the synthesized nanoparticles (Rai et al., 2009). FTIR study was performed to check the existence of such stabilizing agents, and the measurement was done to classify the possible biomolecules present which are responsible for the reduction of the Ag^+^ ions. The nanoparticles formed were not in direct contact not even in aggregate, indicating the stabilization of the nanoparticles by a capping agent as peptide on the surface of nanoparticles. This study was also supported by Ahmad et al. (2003); Chowdhury et al. (2014).

### *In vitro* Antioxidant and Cytotoxic Activities

**Figure 8:**
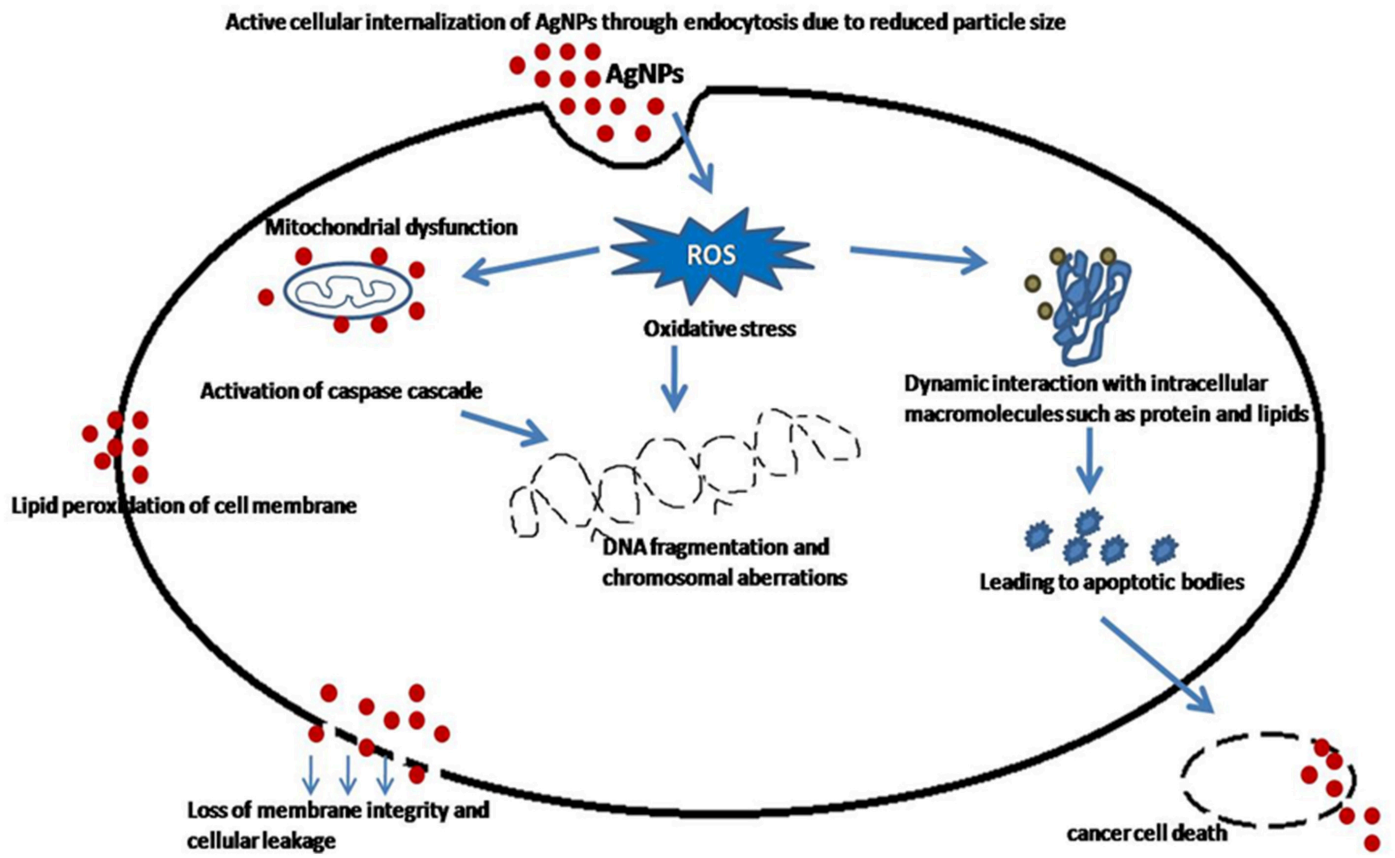
Mechanism involved during cytotoxicity.

It was well-known that SNPs revealed substantial amounts of antioxidant activity which manifested them as potentially drugs (Durán et al., 2010). On the basis of considerable results (Figure 4) of antioxidant activity, it was considered that BSNPs could be effectively used against MCF7, HeLa and HepG2 cancer cell lines. The treated cells have disrupted morphology and have low cell density or number as compared to the control. Toxicity of BSNPs depends upon the shape, size, and the concentration of the nanoparticles. The hypothetical mechanism has been described in Figure 8. These findings suggested that the cell death had occurred which may be due to necrosis or apoptosis. The cytotoxic effects probably occurred due to the interference of Ag NPs with the proper functioning of the protein leading to the change in the cellular chemistry also suggested by Rogers et al. (2008) and Rajpal et al. (2016a,b). Several researchers have suggested that once the Ag NPs penetrates inside the cells, they may cause partial unfolding and cause aggregation of the proteins and also interact with thiol rich enzymes (Morones et al., 2005; Zolghadri et al., 2009). Asharani et al. (2009) and Franco-Molina et al. (2010) reported that chemically synthesized Ag NPs inhibit proliferation of human glioblastoma cells and human breast cancer cells. Sanpui et al. (2011) demonstrated that the chitosan mediated Ag NPs, disrupt the normal cellular function, and also affect its membrane integrity by inducing apoptotic signaling genes of mammalian cells that causes death. According to the recent research Ag NPs have been proven to induce cytotoxic effect due to accumulation in the liver which cause oxidative cell damage (Kim et al., 2009). They also lead to generation of reactive oxygen species producing apoptosis (Carlson et al., 2008; Foldbjerg et al., 2009).

These results are potentially promising and suggest that, we can design a convenient, eco-friendly and cheap method for the synthesis BSNPs with good anticancerous activity. This can open the door for the preparation of suitable pharmaceutical formulation by using these nanoparticles. Taking into account the mobility of Ag NPs into cells as well as outcome in a bioprocess, the risk aspects of the application in large scales and in the environment as well as studies on different biological activities in different fields could be strengthened in future studies.

## Conclusion and Future Prospects

In order to seek for advanced therapeutic concepts for cancer treatment nanosilver formulation has surfaced as a striking option. Our characterization marks the crystalline nature of the particles as well as presence of intrinsic capping and stabilizing protein on the surface of *P. indica*-derived nanoparticles, which further preclude the necessity of further downstream processing, enabling its direct use for anticancerous therapy. The synthesized biogenic Ag NPs displayed impressive cytotoxic potential against three cancer cell lines. The inherent protein cap in these nanoparticles made their entry easier into the cells in comparison to chemically synthesized nanoparticles due to their inherent porosity and therefore larger surface area. The facile synthesis and salient features of this fungus-derived Ag NPs will facilitate their potential applications to the scientific foundation for translational studies in animal.

## Author Contributions

RP and NA conceived and designed the experiments. NA, MF, and MAS performed the experiments. RP, TF, and NA analyzed the data. NA and MF prepared the draft and TF and RP proofread the final draft. All authors approved the final manuscript.

### Conflict of Interest Statement

The authors declare that the research was conducted in the absence of any commercial or financial relationships that could be construed as a potential conflict of interest.

## References

[B1] Ahmad A. Senapati S. Islam Khan M. Kumar R. Sastry M. (2003). Extracellular biosynthesis of monodisperse gold nanoparticles by a novel extremophilic actinomycete, *Thermomonospora* sp. Langmuir 19, 3550–3553. 10.1021/la026772l

[B2] Ahmedin J. Bray F. Center M. M. Ferlay J. Ward E. Forman D. (2011). Global cancer statistics. CA: Cancer J. Clin. 61, 69–90. 10.3322/caac.20107 21296855

[B3] Al-Fatlawi A. A. Ahmad R. A. (2014). Cytotoxicity and pro-apoptotic activity of carvacrol on human breast cancer cell line MCF-7. World J. Pharm. Sci. 2, 1218–1223.

[B4] American Cancer Society (2018). Cancer Facts and Figures 2018. Atlanta: American Cancer Society.

[B5] Arvizo R. R. Bhattacharyya S. Kudgus R. Giri K. Bhattacharya R. Mukherjee P. (2012). Intrinsic therapeutic applications of noble metal nanoparticles: past, present and future. Chem. Soc. Rev. 41, 2943–2970. 10.1039/c2cs15355f 22388295 PMC3346960

[B6] Asharani P. V. Low Kah Mun G. Hande M. P. Valiyaveettil S. (2009). Cytotoxicity and genotoxicity of silver nanoparticles in human cells. ACS Nano 3, 279–290. 10.1021/nn800596w 19236062

[B7] Aziz N. Faraz M. Pandey R. Shakir M. Fatma T. Varma A.. (2015). Facile algae-derived route to biogenic silver nanoparticles: synthesis, antibacterial, and photocatalytic properties. Langmuir 31, 11605–11612. 10.1021/acs.langmuir.5b03081 26447769

[B8] Aziz N. Fatma T. Varma A. Prasad R. (2014). Biogenic synthesis of silver nanoparticles using *Scenedesmus abundans* and evaluation of their antibacterial activity. J. Nanopart. 689419:1–6. 10.1155/2014/689419

[B9] Aziz N. Pandey R. Barman I. Prasad R. (2016). Leveraging the attributes of *Mucor hiemalis*-Derived silver nanoparticles for a synergistic broad-spectrum antimicrobial platform. Front. Microbiol. 7, 1–11. 10.3389/fmicb.2016.01984 28018316 PMC5156874

[B10] Balakumaran M. D. Ramachandran R. Kalaichelvan P. T. (2015). Exploitation of endophytic fungus, *Guignardia mangiferae* for extracellular synthesis of silver nanoparticles and their *in vitro* biological activities. Microbiol. Res. 178, 9–17. 10.1016/j.micres.2015.05.009 26302842

[B11] Bhattacharya R. Mukherjee P. (2008). Biological properties of “naked” metal nanoparticles. Adv. Drug Delivery Rev. 60, 1289–1306. 10.1016/j.addr.2008.03.013 18501989

[B12] Brown K. (2002). Breast cancer chemoprevention: risk-benefit effects of the antioestrogen tamoxifen. Exp. Opin. Drug Saf. 1, 253–267. 10.1093/mutage/gep022 12904141

[B13] Carlson C. Hussein S. M. Schrandetal A. M. (2008). Uniquecellular interaction of silver nanoparticles: size-dependent generation of reactive oxygen species. J. Phy. Chem. B. 112, 13608–13619. 10.1021/jp712087m 18831567

[B14] Chen H. H. W. Kuo M. T. (2017). Improving radiotherapy in cancer treatment: promises and challenges. Oncotarget 8, 62742–62758. 10.18632/oncotarget.18409 28977985 PMC5617545

[B15] Chowdhury S. Basu A. Kundu S. (2014). Green synthesis of protein capped silver nanoparticles from phytopathogenic fungus *Macrophomina phaseolina* (Tassi) goid with antimicrobial properties against multidrug-resistant Bacteria. Nanoscale Res. Lett. 9:365. 10.1186/1556-276X-9-365 25114655 PMC4114801

[B16] Durán N. Marcato P. D. Conti R. D. Alves O. L. Costa F. Brocchi M. (2010). Potential use of silver nanoparticles on pathogenic bacteria, their toxicity and possible mechanisms of action. J. Braz. Chem. Soc. 21, 949–959. 10.1590/S0103-50532010000600002

[B17] Ferlay J. Soerjomataram I. Dikshit R. Eser S. Mathers C. Rebelo M.. (2012). Cancer incidence and mortality worldwide: sources, methods and major patterns in GLOBOCAN. Inter. J. Cancer 136, E359–86. 10.1002/ijc.29210 25220842

[B18] Foldbjerg R. Olesen P. Hougaard M. Dang D. A. Hoffmann H. J. Autrup H. (2009). PVP-coated silver nanoparticles and silver ions induce reactive oxygen species, apoptosis and necrosis in THP-1 monocytes. Toxical. Lett. 190, 156–162. 10.1016/j.toxlet.2009.07.009 19607894

[B19] Franco-Molina M. A. Mendoza-Gamboa E. Sierra-Rivera C. A. Gómez-Flores R. A. Zapata-Benavides P. Castillo-Tello P.. (2010). Antitumor activity of colloidal silver on MCF-7 human breast cancer cells. J. Exp. Clinic. Cancer Res. 29:148. 10.1186/1756-9966-29-148. 21080962 PMC2996348

[B20] Gill S. S. Gill R. Trivedi D. K. Anjum N. A. Sharma K. K. Ansari M. W.. (2016). *Piriformospora indica*: potential and significance in plant stress tolerance. Front. Microbiol. 7:332. 10.3389/fmicb.2016.00332 27047458 PMC4801890

[B21] Hsin Y. H. Chen C. F. Huang S. Shih T. S. Lai P. S. Chueh P. J. (2008). The apoptotic effect of nanosilver is mediated by ROS- and JNK-dependent mechanism involving the mitochondrial pathway in NIH3T3 cells. Toxicol. Lett. 179, 130–139. 10.1016/j.toxlet.2008.04.015 18547751

[B22] Hussain S. M. Hess K. L. Gearhart J. M. Geiss K. T. Schlager J. J. (2005). *In vitro* toxicity of nanoparticles in BRL 3A rat liver cells. Toxicol. In Vitro 1, 975–983 10.1016/j.tiv.2005.06.034 16125895

[B23] Johnston S. R. (1997). Acquired tamoxifen resistance in human breast cancer-potential mechanisms and clinical implications. Anti-Cancer Drugs 8, 911–930. 10.1097/00001813-199711000-00002 9436634

[B24] Kato S. Endoh H. Masuhiro Y. (1995). Activation of the estrogen receptor through phosphorylation by mitogen-activated protein kinase. Science 270, 1491–1494 10.1126/science.270.5241.1491 7491495

[B25] Kaushik U. Joshi S. C. (2015). Silver nanoparticles: green synthesis, optical properties, antimicrobial activity and its mechanism using *Citrus sinensis*. Asian J. Pharma. Clinic. Res. 8, 179–184.

[B26] Kayalvizhi K. Asmathunisha N. Subramanian V. Kathiresan K. (2014). Purification of silver and gold nanoparticles from two species of brown seaweeds (*Padina tetrastromatica* and *Turbinaria ornata*). J. Med. Plants Stu. 2, 32–37.

[B27] Kedar U. Prasanna P. Shidhaye S. Kadam V. (2010). Advances in polymeric micelles for drug delivery and tumor targeting. Nanomed. Nanotechnol. Bio. Med. 6, 714–729. 10.1016/j.nano.2010.05.005 20542144

[B28] Khan I. M. Ahmad A. Miyan L. Ahmad M. Aziz N. (2017). Synthesis of charge transfer complex of chloranilic acid as acceptor with p-nitroaniline as donor: Crystallographic, UV–visible spectrophotometric and antimicrobials. J. Mol. Struct. 1141, 687–6697. 10.1016/j.molstruc.20117.03.050

[B29] Kim S. Choi J. E. Choi J. (2009). Oxidative stress-dependent toxicity of silver nanoparticles in human hepatoma cells. Toxic. in Vitro 23, 1076–1084. 10.1016/j.tiv.2009.06.001 19508889

[B30] Kim Y. S. Song M. Y. Park J. D. Song K. S. Ryu H. R. Chung Y. H.. (2010). Subchronic oral toxicity of silver nanoparticles. Part. Fibre Toxicol. 7, 20–31. 10.1186/1743-8977-7-20 20691052 PMC2928176

[B31] Lee H. Soo P. L. Lin J. Butler M. Allen C. (2007). Polymeric micelles for formulation of anti-cancer drugs, in Nanotech. for Cancer Therapy, eds Amiji M. M (Boca Raton, FL: CRC Press), 317–355.

[B32] Lemire J. A. Harrison J. J. Turner R. J. (2013). Antimicrobial activity of metals: mechanisms, molecular targets and applications. Nat. Rev. Microbiol. 11, 371–384. 10.1038/nrmicro3028 23669886

[B33] Lupu R. Cardillo M. Choetal C. (1996). The significance of heregulin in breast cancer tumor progression and drug resistance. Breast Cancer Res.Treat 38, 57–66. 10.1007/BF01803784 8825123

[B34] Malik M. A. Brien P. O. Revaprasadu N. (2002). A simple route to the synthesis of core/shell nanoparticles of chalcogenides. Chem. Mat. 14, 2004–2010. 10.1021/cm011154w

[B35] Franco-Molina M. A. Mendoza-Gamboa E. Sierra-Rivera C. A. Gómez-Flores R. A. Zapata-Benavides P. Castillo-Tello P. (2010). Antitumor activity of colloidal silver on MCF-7 human breast cancer cells. J. Exp. Clinic. Cancer Res. 29:148. 10.1186/1756-9966-29-148 21080962 PMC2996348

[B36] Morones J. R. Elechiguerra J. L. Camacho A. Holt K. Kouri J. B. Ramírez J. T.. (2005). The bactericidal effect of silver nanoparticles. Nanotechnol 16, 23–46. 10.1088/0957-4484/16/10/059 20818017

[B37] Ngo Y. H. Li D. Simon G. P. Garnier G. (2011). Paper surfaces functionalized by nanoparticles. Adv. Colloid Interface Sci. 163, 23–38. 10.1016/j.cis.2011.01.004 21324427

[B38] Ong C. Lim J. Z. Ng C. T. Li J. J. Yung L. Y. Bay B. H. (2013). Silver nanoparticles in cancer: therapeutic efficacy and toxicity. Curr. Med. Chem. 20, 772–781. 10.2174/0929867311320060003 23298139

[B39] Pandey S. Goswami G. K. Nanda K. K. (2012). Green synthesis of biopolymer silver nanoparticle nanocomposite: an optical sensor for ammonia detection. Int. J. Biol. Macromol. 51, 583–589. 10.1016/j.ijbiomac.2012.06.033 22750580

[B40] Prabhu S. Poulose E. K. (2012). Silver nanoparticles: mechanism of antimicrobial action, synthesis, medical applications, and toxicity effects. Int. Nano. Lett. 2:32 10.1186/2228-5326-2-32

[B41] Prasad R. (2014). Synthesis of silver nanoparticles in photosynthetic plants. J. Nanopart. 2014:963961 10.1155/2014/963961

[B42] Prasad R. Kamal S. Sharma P. K. Oelmueller R. Varma A. (2013). Root endophyte *Piriformospora indica* DSM 11827 alters plants morphology, enhances biomass and antioxidant activity of medicinal plant *Bacopa monniera*. J. Basic Microbiol. 53, 1016–1024. 10.1002/jobm.201200367 23681554

[B43] Prasad R. Pandey R. Barman I. (2016). Engineering tailored nanoparticles with microbes: quo vadis? WIREs Nanomed. Nanobiotechnol. 8, 316–30. 10.1002/wnan.1363 26271947

[B44] Premasudha P. Venkataramana M. Abirami M. Vanathi P. Krishna K. Rajendran R. (2015). Biological synthesis and characterization of silver nanoparticles using *Eclipta alba* leaf extract and evaluation of its cytotoxic and antimicrobial potential. Bull. Mater. Sci. 38, 965–973. 10.1007/s12034-015-0945-5

[B45] Rai M. Yadav A. Gade A. (2009). Silver nanoparticles as a new generation of antimicrobials. Biotechnol. Adv. 27, 76–83. 10.1016/j.biotechadv.2008.09.002 18854209

[B46] Rajpal K. Aziz N. Prasad R. Varma R. G. Varma A. (2016a). Evaluating bionanoparticle infused fungal metabolites as a novel antimicrobial agent. J. Adv. Pharma. Tech. Res. 7, 42–46. 10.4103/2231-4040.184593 27429931 PMC4932805

[B47] Rajpal K. Aziz N. Prasad R. Varma R. G. Varma A. (2016b). Extremophiles as biofactories of novel antimicrobials and cytotoxics- An assessment of bioactive properties of six fungul species inhabiting ran of Kutch, India. Indian J. Sci. Tech. 9, 1–9. 10.17485/ijst/2016/v9i24/89609

[B48] Ray S. Sarkar S. Kundu S. (2011). Extracellular biosynthesis of silver nanoparticles using the mycorrhizal mushroom *Tricholoma crassum* (Berk.) Sacc: its antimicrobial activity against pathogenic bacteria and fungus, including multidrug resistant plant and human bacteria. Dig J. Nanomater. Biostruct. 6, 1289–1299

[B49] Rogers J. V. Parkinson C. V. Choi Y. W. Speshock J. L. Hussain S. M. (2008). A preliminary assessment of silver nanoparticle inhibition of monkeypox virus plaque formation. Nanoscale Res. Lett. 3, 129–133. 10.1007/s11671-008-9128-2

[B50] Sanpui P. Chattopadhyay A. Ghosh S. S. (2011). Induction of apoptosis in cancer cells at low silver nanoparticle concentrations using chitosan nanocarrier. ACS App. Mat. Int. 3, 218–228. 10.1021/am100840c 21280584

[B51] Senapati S. Mahanta A. K. Kumar S. Maiti P. (2018). Controlled drug delivery vehicles for cancer treatment and their performance. Signal Transduct. Target Ther. 3:7. 10.1038/s41392-017-0004-3 29560283 PMC5854578

[B52] Shin K. S. Choi J. Y. Park H. J. Jang Kim K. (2009). Facile synthesis and catalytic application of silver-deposited magnetic nanoparticles. Catalysis Letters. 133, 1–7. 10.1007/s10562-009-0124-7

[B53] Siddhanta S. Paidi S. K. Bushley K. Prasad R. Barman I. (2017). Exploring morphological and biochemical linkages in fungal growth with label-free light sheet microscopy and Raman spectroscopy. ChemPhysChem. 18, 72–78. 10.1002/cphc.201601062 27860053

[B54] Siegel R. L. Miller K. D. Jemal A. (2016). Cancer statistics, 2016. Ca-Cancer J. Clin. 66, 7–30. 10.3322/caac.21332 26742998

[B55] Singh R. P. Ramarao P. (2012). Cellular uptake, intracellular trafficking, and cytotoxicity of silver nanoparticles. Toxicol. Lett. 213, 249–259. 10.1016/j.toxlet.2012.07.009 22820426

[B56] Sonoda E. Sasaki M. S. Buerstedde J. M. Bezzubova O. Shinohara A. Ogawa H. (1998). Rad51- deficient vertebrate cells accumulate chromosomal breaks prior to cell death. EMBO J. 15, 598–608. 10.1093/emboj/17.2.598 PMC1170409 9430650

[B57] Sukirtha R. Priyanka K. M. Antony J. J. Kamalakkannan S. Thangam R. Gunasekaran P. (2012). Cytotoxic effect of green synthesized silver nanoparticles using *Melia azedarach* against *in vitro* HeLa cell lines and lymphoma mice model. Process Biochem. 47, 273–279. 10.1016/j.procbio.2011.11.003

[B58] Thakkar K. N. Mhatre S. S. Parikh R. Y. (2010). Biological synthesis of metallic nanoparticles. Nanomedicine 6, 257–262. 10.1016/j.nano.2009.07.002 19616126

[B59] Varma A. Bakshi M. Lou B. Hartmann A. Oelmueller R. (2012). Piriformospora indica: A novel plant growth-promoting mycorrhizal fungus. Agri. Res. 1, 117–131. 10.1007/s40003-012-0019-5

[B60] Varma A. Verma S. Sudha S. N. Butehorn B. Franken P. (1999). Piriformospora indica, a cultivable plant-growth-promoting root endophyte. Appl. Environ. Microbiol. 65, 2741–2744. 10347070 10.1128/aem.65.6.2741-2744.1999 PMC91405

[B61] Yang X. Du Y. Li D. Lv Z. Wang E. (2011). One-step synthesized silver micro-dendrites used as novel separation mediums and their applications in multi-DNA analysis. Chem. Commun. 47, 10581–10583. 10.1039/c1cc11374g 21512713

[B62] Yoon K. Hoon B. Park J. H. Hwang J. (2007). Susceptibility constants of *Escherichia coli* and *Bacillus subtilis* to silver and copper nanoparticles. Sci. Total Environ. 373, 572–575. 10.1016/j.scitotenv.2006.11.007 17173953

[B63] Zeng H. Chen W. Zheng R. Zhang S. Ji S. J. Zou X.. (2018). Changing cancer survival in China during 2003–15: a pooled analysis of 17 population-based cancer registries. Lancet 6, 555–567, 10.1016/S2214-109X(18)30127-X 29653628

[B64] Zhou X. He X. He D. Wang K. Qin D. (2011). Biosensing technologies for *Mycobacterium tuberculosis* detection: status and new developments. Clin. Dev. Immunol. 2011:193963. 10.1155/2011/193963 21437177 PMC3061460

[B65] Zolghadri S. Saboury A. Golestani A. Divsalar A. Rezaei-Zarchi S. Moosavi-Movahedi A. (2009). Interaction between silver nanoparticle and bovine hemoglobin at different temperatures. J. Nanopar. Res. 11, 1751–1758. 10.1007/s11051-008-9538-1

